# Weight Status and Psychological Distress in a Mediterranean Spanish Population: A Symmetric U-Shaped Relationship

**DOI:** 10.3390/nu6041662

**Published:** 2014-04-21

**Authors:** Elena Villalobos Martínez, Mario Gutiérrez-Bedmar, Antonio García-Rodríguez, Alberto Mariscal, Carlos Muñoz-Bravo, Joaquín Fernández-Crehuet Navajas

**Affiliations:** 1 Community Mental Health Service, Antequera Hospital, Avda. Poeta Muñoz Rojas, s/n, Antequera, Málaga 29200, Spain; E-Mail: zahara_110@yahoo.es (E.V.M.); 2 Department of Preventive Medicine and Public Health, University of Málaga, Boulevard Louis Pasteur, 32, Málaga 29071, Spain; E-Mails: antoniogr@uma.es (A.G.-R); mariscal@uma.es (A.M.); carlosmb@uma.es (C.M.-B.); crehuet@uma.es (J.F.-C.N.)

**Keywords:** body mass index, psychological distress, SCL-90-R, obesity, underweight

## Abstract

Psychological disorders in people with extreme weight (low weight or obesity) should be taken into consideration by health professionals in order to practice an effective treatment to these patients. This study evaluates the association between body mass index (BMI) and psychological distress in 563 inhabitants of Málaga (South of Spain). Participants were classified in four categories of BMI: Underweight (BMI <18.5 Kg/m^2^), Normal weight (BMI 18.5–24.99 Kg/m^2^), Overweight (BMI 25.0–29.99 Kg/m^2^) and Obesity (BMI >30 Kg/m^2^). Psychological distress was measured with the Spanish version of the Derogatis’ Symptoms Checklist Revised (SCL-90-R). We observed a symmetric U-shaped relationship between weight status and psychological distress in all SCL-90-R dimensions (*p* for quadratic trend <0.001) for both men and women. Participants with extreme weight showed the worst psychological status, and participants with normal weight exhibited the best. We found no statistically significant differences between underweight and obese participants in 9 of the 10 SCL-90-R dimensions analyzed among men, and in 8 of the 10 dimensions among women. Underweight and obese participants showed no gender differences in psychological distress levels. Psychological treatment of Mediterranean people with extreme weight, should consider underweight and obese patients at the same level of psychological distress.

## 1. Introduction

Relationship between body mass index (BMI) and psychological status has been examined in a significant number of studies [1,2,3], most of them focused on overweight and obese individuals. These studies generally have observed a positive association between the two variables. Nevertheless, among those studies that considered all the BMI categories (from underweight to obesity), a U- or J-shaped relationship have been observed [4,5,6,7,8,9].

The knowledge about the burden of psychological disease in people with extreme weight (underweight or obesity) should be taken into consideration by health professionals in order to practice an effective treatment to these patients. Two examples of the above are the psychological screening of bariatric surgery candidates, which optimizes their postoperative outcomes [10,11,12], or the association between the absence of psychopathology and weight recovery in females with anorexia nervosa [13]. From a practice point of view, it is important to know whether underweight people have worse psychological status than obese people or *vice versa*, because this issue is not clear yet [4,5,7,8,9].

The measurement of psychological distress is not “homogeneous” in those studies focusing on the relationship between BMI and psychological status. Most of them are centered on depression, anxiety or mood disorders as a dichotomous outcome variable [4,6,9,14,15,16,17]. Another studies use cut-off points of psychological distress scales to classify participants according to their psychological distress level [7,8,18,19]. Although this categorization of the outcome variable entails a loss of information about the level and variability of psychological distress, we have found only one study on the topic that uses scores of psychological distress scales as numerical outcome variables [5]. In order to obtain as much information as possible about the level of psychological distress across BMI, it would be convenient to use a comprehensive psychological scale, covering the main psychopathological symptoms.

A Mediterranean-style diet has been associated with a better psychological status [20,21,22] and less incidence of overweight or obesity [23,24]. It would be interesting to verify whether relationships between psychological distress and BMI observed in other populations, will be maintained in a Mediterranean population because, to our knowledge, such study has not been published before.

Thus, we aimed to evaluate the shape of the relationship between BMI and psychological distress in a Mediterranean Spanish population with a comprehensive psychological scale. In addition, we wanted to know what weight status as well as what sex, had higher levels of psychological distress in our population.

## 2. Materials and Methods

### 2.1. Population and Sample

The study population consisted of inhabitants of Málaga (South of Spain) aged between 18 and 65 years, who were assigned to a health center within Málaga Health District. To get a sample from this population, a systematic random sample was drawn. The sample was non-proportional stratified by BMI categories in order to include enough participants in extreme categories of BMI. Between January and December of 2011, a total of 563 participants were recruited from those who came to his primary care physician with a disease not related with body weight or psychological status. Inclusion criteria were men or women aged between 18 and 65 years, who accepted to participate in the study and signed the Informed Consent Form. Exclusion criteria were presence of a handicap that prevents giving a reliable answer, history of psychiatric disorder during the last two years, intake of drugs related to weight change, and any change in BMI due to: metabolic or neuroendocrine etiology, genetic malformation syndromes, lipomathosis or lipodystrophy.

The Ethics Committee of the Faculty of Medicine of de University of Málaga approved this study.

### 2.2. Measures

Height and weight measures were obtained with the same instruments by trained study staff. BMI was calculated as weight (Kg) divided by square of height in meters (m^2^). According to WHO international classification [25], participants were classified into one of four groups: Underweight (BMI <18.5 kg/m^2^), Normal weight (BMI 18.5–24.99 Kg/m^2^), Overweight (BMI 25.0–29.99 Kg/m^2^) and Obesity (BMI >30 Kg/m^2^). Although categorization of a variable implies loss of information [26], almost all the studies focusing on the relationship between BMI and psychological status used BMI as a categorical variable (with the WHO classification) rather than a continuous variable. Therefore, this categorization of BMI allows us to compare our results with those on the same topic.

Psychological distress was rated using the Spanish version of the Symptoms Checklist 90-Revised (SCL-90-R) [27]. The SCL-90-R is a self-report inventory containing 90 items. Participants were instructed to indicate how much distress each item has caused during the last seven days (including the interview day), on a five-point scale ranging from 0 (not at all) to 4 (extremely). This instrument reports nine primary symptoms dimensions namely somatization, obsessive-compulsive, interpersonal sensitivity, depression, anxiety, hostility, phobic anxiety, paranoid ideation and psychoticism. In addition, it includes the Global Severity Index (GSI), which is the mean score of all items. The GSI is considered the single best indicator of current distress level and should be utilized when a single summary measure is required. So, we considered 10 dimensions of the questionnaire: 9 primary symptoms and the GSI.

We collected additional information regarding participants’ age, gender, education level (no studies, primary, secondary, university), having a paid work (yes/no), living alone (yes/no), origin (rural/urban), family history of obesity (yes/no), and family history of psychiatric disease (yes/no). Participants were considered with family history of psychiatric disease if their father, mother or brethren received psychiatric treatment four or more times in the last five years. These variables were used to adjust for the possible confounding effect.

### 2.3. Data Analysis

First, we described the sample by studying variables according to BMI categories. The association between BMI and the study variables was assessed using the Chi-square test. The association between two dichotomous variables was assessed using the Fisher exact test.

Adjusted mean scores of SCL-90-R dimensions were estimated using General Linear Models. In the multivariate analysis, we included all variables whose bivariate test was significant (defined as a two tailed *p* value ≤0.05) and those we considered scientifically relevant according to the revised references reviewed. For each SCL-90-R dimension, assessment of interaction between BMI and gender was performed by adding a multiplicative interaction term (BMI × Gender) in the multivariate models. After confirming the presence of interaction and based on literature [4,18], we carried out all the analysis separately for men and women.

Statistical differences in mean scores between BMI categories were determined by ANCOVA. Whether these adjusted means were statistically significantly different (*p* ≤ 0.05) was evaluated post hoc using the Bonferroni correction. The presence of a U-shaped relationship between BMI categories and SCL-90-R dimensions was evaluated with polynomial contrasts (quadratic trend). Partial eta squared (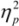) were reported as measures of effect size. Following Cohen’s criteria [28], we considered small effect 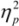 = 0.0099; medium effect 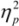 = 0.0588 and large effect 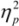 = 0.1379.

All the analyses were conducted with SPSS 20.0 for Mac (IBM Corp., Armonk, NY, USA).

## 3. Results

Of the total sample (*N* = 563), 78 (13.9%) were underweight, 142 (25.2%) had a normal weight, 170 (30.2%) had overweight and 173 (30.7%) were obese.

Table 1 shows the distribution of the socio demographic variables and their association with BMI categories. We found statistical association with age (*p* < 0.001), education level (*p* < 0.001), paid work (*p* < 0.001), family history of obesity (*p* < 0.001) and family history of psychiatric disease (*p* < 0.001), and we did not find statistical association with gender, live alone and origin.

Concerning age, we found that the higher BMI, the higher age. Pearson’s coefficient correlation between both variables (considered as continuous) was *r* = 0.383 (*p* < 0.001) (data not shown). Educational level differs among groups. We have found a high percentage of people without studies in the obese group (36.6%) and none in the underweight group. Regarding paid work, there are no statistical differences between the normal weight and overweight group (Fishers’ exact test *p*-value = 0.302), and between underweight and obese group (Fishers’ exact test *p*-value = 0.108). The presence of family history of obesity is highest in the obese group (23.1%) and lowest in the normal weight group (4.9%). When we compared the underweight with the overweight group, we found no statistical differences (Fishers’ exact test *p*-value = 1). We found a similar association between weight status and family history of psychiatric disease. The obese group had the highest percentage of family history of psychiatric disease (28.3%), and the normal weight group had the lowest percentage (9.2%). Again, when we compared the underweight with the overweight group, we found no statistical differences (Fishers’ exact test *p*-value = 0.562).

**Table 1:** Socio demographic variables and their association with body mass index.

Variable	Value	Total		Underweight		Normal weight		Overweight		Obese		*p* ^1^
		N	(%)	N	(%)	N	(%)	N	(%)	N	(%)	
Gender	Male	235	(41.7)	37	(47.4)	63	(44.4)	67	(39.4)	68	(39.3)	0.523
	Female	328	(58.3)	41	(52.6)	79	(55.6)	103	(60.6)	105	(60.7)	
Age	15–24	79	(14.1)	52	(66.7)	929593015	(6.3)	18	(10.6)	0	(0)	<0.001
	25–34	15	(20.5)	17	(21.8)	29	(20.4)	44	(25.9)	25	(14.5)	
	35–44	191	(34.0)	3	(3.8)	59	(41.5)	52	(30.6)	77	(44.8)	
	45–54	115	(20.5)	6	(7.7)	30	(21.1)	37	(21.8)	42	(24.4)	
	55–64	62	(11.0)	0	(0)	15	(10.6)	19	(11.2)	28	(16.3)	
Education	No studies	74	(14.6)	0	(0)	3	(2.5)	18	(11.2)	53	(36.6)	<0.001
	Primary	183	(36.2)	35	(44.9	46	(37.7)	53	(32.9)	49	(33.8)	
	Secondary	162	(32.0)	36	(46.2)	41	(33.6)	60	(37.3)	53492518	(17.2)	
	University	87	(17.2)	7	(9.0)	32	(26.2)	30	(18.6)	18	(12.4)	
Paid work	Yes	235	(41.7)	13	(16.7)	85	(59.9)	91	(53.5)	46	(26.6)	<0.001
	No	328	(58.3)	65	(83.3)	57	(40.1)	79	(46.5)	127	(73.4)	
Live alone	Yes	79	(14.0)	11	(14.1)	11	(7.7)	25	(14.7)	32	(18.5)	0.056
	No	484	(86.0)	67	(85.9)	131	(92.3)	145	(85.3)	141	(81.5)	
Origin	Rural	74	(13.1))	6	(7.7)	16	(11.3)	20	(11.8)	32	(18.5)	0.069
	Urban	489	(86.9)	72	(92.3)	126	(88.7)	150	(88.2)	141	(81.5)	
FH ^2^ Obesity	Yes	84	(14.9)	12	(15.4)	7	(4.9)	25	(14.7)	40	(23.1)	<0.001
	No	579	(85.1)	66	(84.6)	135	(95.1)	145	(85.3)	133	(76.9)	
FH ^2^ Psychiatric Disease	Yes	98	(17.4)	13	(16.7)	13	(9.2)	23	(13.5)	49	(28.3)	<0.001
	No	465	(82.6)	65	(83.3)	129	(90.8)	147	(86.5)	124	(71.7)	

^1^ Chi-square test; ^2^ FH = Family History of

When interaction by gender was analyzed, we found statistically significant interaction between gender and BMI for each SCL-90-R primary symptoms dimensions (*p* < 0.001) and for GSI (*p* < 0.049) (data not shown).

**Figure 1:**
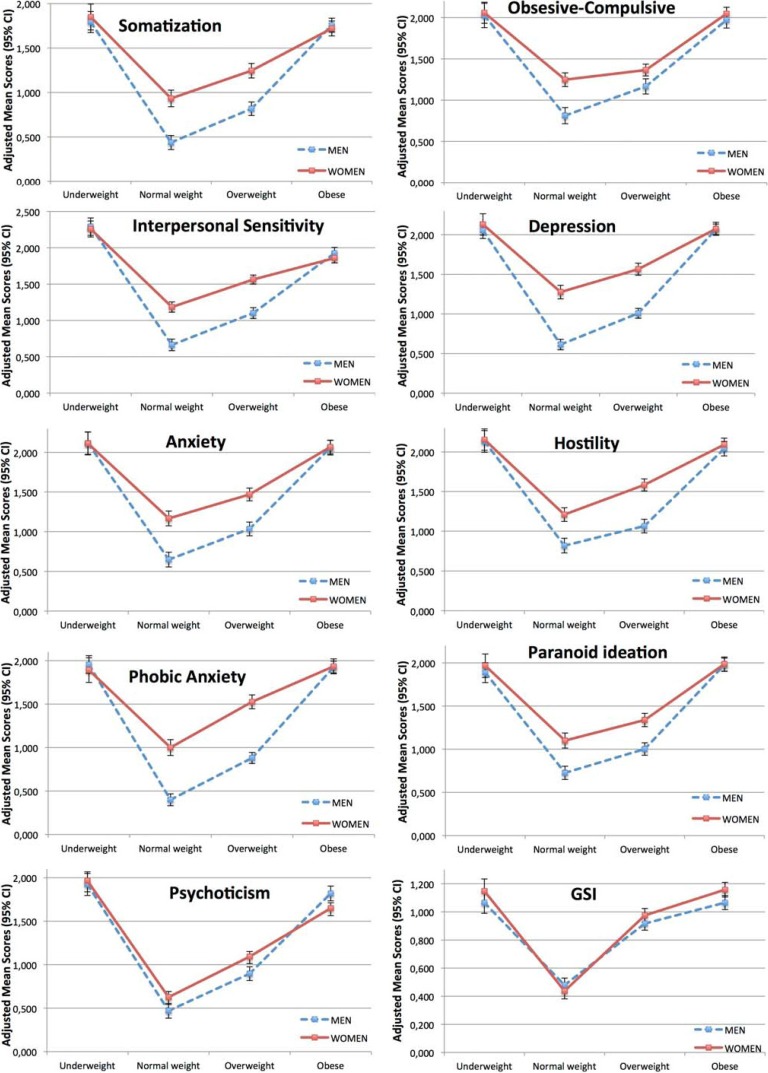
Multivariate adjusted mean scores (with 95% CIs) for SCL-90-R primary symptoms dimensions and global severity index (GSI) by gender in each BMI category. CI, confidence interval.

Table 2 shows the adjusted mean scores of the psychological distress dimensions (including GSI) by BMI categories among men. We found statistical association between BMI categories and all SCL-90-R dimensions (*p* < 0.001). The results of the polynomial trend analyses indicated a significant positive quadratic effect between categorical BMI and all SCL-90-R dimensions (*p* for quadratic trend <0.001). This indicates that each SCL-90-R dimension (including GSI) shows a positive quadratic trend (U-shaped trend) in the association with BMI categories among men. Figure 1 shows graphically the symmetric U-shape relationship between BMI status and all SCL-90-R dimensions among men. Partial eta squared showed a large effect size for all SCL-90-R dimensions. The higher effect size was for phobic anxiety (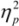 = 0.839) and the lowest was for GSI (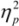 = 0.584).

**Table 2:** Multivariate adjusted mean scores (95% confidence interval) on the SCL-90-R questionnaire according to body mass index categories among men.

SCL-90-R dimensions	Underweight	Normal weight	Overweight	Obese	*p*-value (ANCOVA)	*p* for quadratic trend	Partial eta squared ( 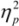 )
Somatization ^1^	1.792 *^#^ (1.673–1.911)	0.437 (0.358–0.516)	0.817 * (0.741–0.894)	1.758 *^#^ (1.680-1.837)	<0.001	<0.001	0.771
Obsesive-compulsive ^2^	2.026 *^#^ (1.879–2.173)	0.813 (0.715–0.911)	1.167 * (1.075–1.258)	1.972 *^#^ (1.874–2.070)	<0.001	<0.001	0.601
Interpersonal sensitivity ^2^	2.291 *^#§^ (2.171–2.410)	0.663 (0.583–0.743)	1.101 * (1.026–1.175)	1.925 *^#^ (1.845–2.005)	<0.001	<0.001	0.759
Depression ^2^	2.053 *^#^ (1.953–2.153)	0.614 (0.548–0.681)	1.008 * (0.946–1.070)	2.068 *^#^ (2.001–2.135)	<0.001	<0.001	0.834
Anxiety ^2^	2.116 *^#^ (1.976–2.256)	0.649 (0.556–0.743)	1.033 * (0.946–1.120)	2.059 *^#^ (1.966–2.152)	<0.001	<0.001	0.714
Hostility ^2^	2.132 *^#^ (1.995–2.269)	0.820 (0.729–0.912)	1.064 * (0.978–1.149)	2.040 *^#^ (1.949–2.131)	<0.001	<0.001	0.679
Phobic anxiety ^2^	1.955 *^#^ (1.853–2.058)	0.399 (0.331–0.468)	0.880 * (0.816–0.944)	1.926 *^#^ (1.858–1.995)	<0.001	<0.001	0.839
Paranoid ideation ^2^	1.887 *^#^ (1.772–2.003)	0.726 (0.649–0.803)	1.002 * (0.930–1.074)	1.978 *^#^ (1.901–2.056)	<0.001	<0.001	0.737
Psychoticism ^2^	1.923 *^#^ (1.796–2.050)	0.470 (0.385–0.555)	0.897 * (0.818–0.976)	1.819 *^#^ (1.734–1.903)	<0.001	<0.001	0.734
Global Severity Index ^2^	1.065 *^#^ (0.990–1.140)	0.479 (0.429–0.528)	0.917 * (0.870–0.963)	1.066 *^#^ (1.016–1.116)	<0.001	<0.001	0.584

^1^ Adjusted by age; ^2^ Adjusted by age, paid work, studies, family history of obesity and family history of psychiatric disease; * Statistically significantly (*p* < 0.05) higher than Normal weight (Bonferroni post-test correction); ^#^ Statistically significantly (*p* < 0.05) higher than Overweight (Bonferroni post-test correction); ^§^ Statistically significantly (*p* < 0.05) higher than Obese (Bonferroni post-test correction).

The normal weight group showed lower (*i.e.*, better) SCL-90-R adjusted mean scores than the other groups in all dimensions and GSI (*p* < 0.05). Overweight men had lower SCL-90-R adjusted mean scores than underweight and obese men in all dimensions and GSI (*p* < 0.05). When we compared SCL-90-R adjusted mean scores between underweight and obese men, we only found statistically significant differences in interpersonal sensitivity (adjusted mean in underweight group = 2.291; adjusted mean in obese group = 1.972; *p* < 0.05).

If we focus on the nine SCL-90-R dimensions (*i.e.*, excluding GSI), underweight men achieved the highest adjusted mean score in interpersonal sensitivity (2.291), and the lowest in somatization (1.792). In normal weight men, the highest mean score was for hostility (0.820), and the lowest was for phobic anxiety (0.399). Concerning overweight men, the highest mean score was for obsessive-compulsive (1.167) and the lowest was for somatization (0.817). Finally, obese men achieved the highest adjusted mean score in depression (2.068), and the lowest in somatization (1.758).

Concerning GSI, the highest adjusted mean score was found in obese men (1.132), and the lowest in normal weight men (0.479).

Table 3 shows the adjusted mean scores of the psychological distress dimensions (including GSI) by BMI categories among women. We found statistical association between BMI categories and all SCL-90-R dimensions (*p* < 0.001). Results of the polynomial trend analyses indicated a significant positive quadratic effect between categorical BMI and all SCL-90-R dimensions (*p* for quadratic trend <0.001). This indicates that each SCL-90-R dimension (including GSI), shows a positive quadratic trend (U-shaped trend) in the association with BMI categories among women. Figure 1 shows graphically the symmetric U-shape relationship between BMI status and all SCL-90-R dimensions among women. Partial eta squared showed a large effect size for all SCL-90-R dimensions. The higher effect size was for psychoticism (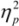 = 0.690) and the lowest was for somatization (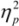 = 0.410).

**Table 3:** Multivariate adjusted mean scores (95% confidence interval) on the SCL-90-R questionnaire according to body mass index levels among women.

SCL-90-R dimensions	Underweight	Normal weight	Overweight	Obese	*p*-value (ANCOVA)	*p* for quadratic trend	Partial eta squared ( 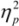 )
Somatization ^1^	1.849 * ^#^ (1.703–1.994)	0.933 (0.840–1.027)	1.245 * (1.163–1.327)	1.720 *^#^ (1.636–1.803)	<0.001	<0.001	0.401
Obsesive-compulsive ^2^	2.059 *^#^ (1.931–2.187)	1.248 (1.165–1.330)	1.365 (1.293–1.437)	2.047 *^#^ (1.969–2.124)	<0.001	<0.001	0.472
Interpersonal sensitivity ^2^	2.259 *^#§^ (2.149–2.370)	1.184 (1.113–1.255)	1.563 * (1.501–1.625)	1.858 *^#^ (1.792–1.925)	<0.001	<0.001	0.510
Depression ^2^	2.130 *^#^ (1.995–2.266)	1.276 (1.190–1.363)	1.565 * (1.489-1.641)	2.075 *^#^ (1.993–2.157)	<0.001	<0.001	0.410
Anxiety ^2^	2.113 *^#^ (1.968–2.258)	1.165 (1.072–1.258)	1.468 * (1.387–1.550)	2.067 *^#^ (1.980–2.155)	<0.001	<0.001	0.437
Hostility ^2^	2.155 *^#^ (2.020–2.290)	1.209 (1.123–1.296)	1.583 * (1.507–1.659)	2.091 *^#^ (2.009–2.172)	<0.001	<0.001	0.453
Phobic anxiety ^2^	1.891 *^#^ (1.749–2.033)	1.000 (0.909–1.091)	1.526 * (1.446-1.606)	1.934 *^#^ (1.848–2.020)	<0.001	<0.001	0.428
Paranoid ideation ^2^	1.967 *^#^ (1.831–2.103)	1.100 (1.013–1.188)	1.338 * (1.261–1.415)	1.986 *^#^ (1.904–2.069)	<0.001	<0.001	0.458
Psychoticism ^2^	1.965 *^#§^ (1.862–2.069)	0.626 (0.560–0.693)	1.092 * (1.033–1.150)	1.649 *^#^ (1.586–1.711)	<0.001	<0.001	0.690
Global Severity Index ^2^	1.148 *^#^ (1.062–1.235)	0.437 (0.381–0.492)	0.975 * (0.926–1.023)	1.158 *^#^ (1.106–1.210)	<0.001	<0.001	0.560

^1^ Adjusted by age; ^2^ Adjusted by age, paid work, studies, family history of obesity and family history of psychiatric disease; * Statistically significantly (*p* < 0.05) higher than Normal weight (Bonferroni post-test correction); ^#^ Statistically significantly (*p* < 0.05) higher than Overweight (Bonferroni post-test correction); ^§^ Statistically significantly (*p* < 0.05) higher than Obese (Bonferroni post-test correction).

Women with normal weight showed lower SCL-90-R adjusted mean scores than underweight and obese women in all dimensions and GSI (*p* < 0.05). With the exception of obsessive-compulsive dimension, women with normal weight had lower adjusted mean scores than women with overweight in all dimensions and GSI (*p* < 0.05). Women with overweight had lower SCL-90-R adjusted mean scores than underweight and obese women in all dimensions and GSI (*p* < 0.05). When we compared SCL-R adjusted mean scores between underweight and obese women, we found statistically significant differences in interpersonal sensitivity (adjusted mean in underweight group = 2.259; adjusted mean in obese group = 1.858; *p* < 0.05) and psychoticism (adjusted mean in underweight group = 1.965; adjusted mean in obese group = 1.649; *p* < 0.05).

If we focus on the nine SCL-90-R dimensions (*i.e.*, excluding GSI), underweight women achieved the highest adjusted mean score in interpersonal sensitivity (2.259), and the lowest in somatization (1.849). In normal weight women, the highest mean score was for depression (1.276), and the lowest was for psychoticism (0.626). Concerning overweight women, the highest mean score was for hostility (1.583) and the lowest was for psychoticism (1.092). Finally, obese women achieved the highest adjusted mean score in hostility (2.091), and the lowest in psychoticism (1.649).

Concerning GSI, the highest adjusted mean score was found in obese women (1.158), and the lowest in normal weight women (0.437).

**Figure 2:**
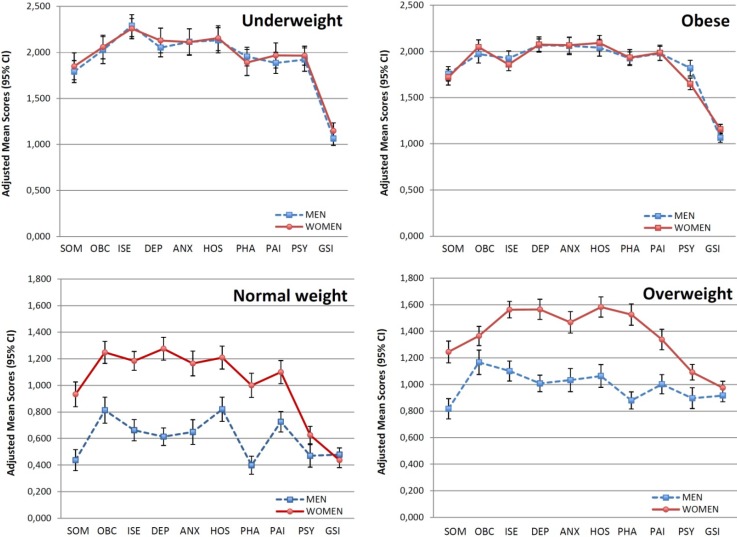
Multivariate adjusted SCL-90-R mean scores (with 95% CIs) for underweight, normal weight, overweight and obese by gender. CI, confidence interval; SOM, somatization; OBC, obsessive-compulsive; ISE, interpersonal sensitivity; DEP, depression; ANX, anxiety; HOS, hostility; PHA, phobic anxiety; PAI, paranoid ideation; PSY, psychoticism; GSI, global severity index.

When we compared SCL-90-R adjusted mean scores between men and women (Figure 2), we found that women with normal weight showed statistically significantly (*p* < 0.05) higher adjusted mean scores than men with normal weight in the nine SCL-90-R primary symptom dimensions, but not in the GSI. Overweight women also showed statistically significantly (*p* < 0.05) higher adjusted mean scores than men with overweight in the nine primary symptom dimensions, but not in the GSI. Among obese participants, we have only found that men showed statistically significantly (*p* < 0.05) higher adjusted mean score than women in psychoticism dimension. Finally, we found no statistically significant differences in SCL-90-R adjusted mean scores between men and women with underweight.

## 4. Discussion

In this cross-sectional study, we observe a symmetric U-shaped relationship between weight status and psychological distress for both men and women. Participants with extreme weight, *i.e.*, underweight or obese, showed the worst psychological status, and participants with normal weight exhibited the best. We found no statistically significant differences between underweight and obese participants in 9 of the 10 SCL-90-R dimensions analyzed among men, and in 8 of the 10 dimensions among women. By sexes, women showed a worse psychological status than men either in normal weight group or in overweight group. These differences disappear when we compared psychological status between women and men either underweight or obese.

The non-linear association between BMI and morbi-mortality is well known. U- or J- shaped relationships between BMI and mortality have been described in several studies [29,30,31]. Recent studies have also described a U- or J- shaped association between BMI and physical morbidity [32,33]. Concerning mental health, the shape of the association between BMI and mental disorders has been specifically analyzed in three studies. McCrea *et al.* [4] used cubic splines to study the relationship between BMI and common mental disorders in 7043 English adults. They found that, in young men, the relationship was U-shaped. Kelly *et al.* [5] analyzed mental ill-health by deciles of BMI in a large sample (*N* = 42,807) of Australians. They observed a clear non-linear association and suggest a J-shaped relationship between BMI and mental ill-health. Finally, de Wit *et al.* [6], based on a sample of 43,534 individuals from the Netherlands, found a very significant U-shaped association between BMI and depression. Based on these works, we aimed to verify the U- or J-shape association in our sample, and our results support a clear U-shape relationship between BMI and psychological distress.

In our view, one of the main finding of this work was the symmetry observed between underweight and obese participants (Figure 1). Almost all the works focused on studying overweight and obese individuals, found positive associations between BMI and different measurements of psychological distress [3,15,18,34,35,36,37,38]. Nevertheless, among those studies that considered all the BMI categories (from underweight to obese), it is not clear what is the category with a worse psychological status (underweight or obese). Some studies establish that underweight people have a worse psychological status than obese people. On this line, Zhao *et al.* [7] examined the associations of BMI with serious psychological distress in a large sample of U.S. adults (*N* = 153,865) and they found that, in men, the age-adjusted prevalence of serious psychological distress was higher in underweight than in obese. In women, they found that underweight participants had higher adjusted prevalence of serious psychological distress than those with a BMI between 30 and 40 Kg/m^2^, but lower than women with a BMI > 40 Kg/m^2^. In a cross-sectional study of 17,253 Australians, Atlantis *et al.* [8] found that medium and high psychological distress prevalence was higher in underweight than in obese participants. A more recent cross-sectional study on 7,043 English adults [4] found that underweight participants had higher adjusted prevalence of any common mental disorder than those with a BMI between 30 and 40 Kg/m^2^, but lower than participants with a BMI > 40 Kg/m^2^. By other hand, there are studies which show that obese had a worse psychological status compared to underweight people. In a sample of 41,654 U.S. adults, Petry *et al.* [9] found that the lifetime prevalence of any mood disorder and any anxiety disorder was higher for obese than for underweight people. Kelly *et al.* [5] studied mental ill-health by deciles of BMI in a large sample (*N* = 42,807) of Australians, and they observed statistically significantly greater odds of mental ill-health only in the obese and not in the underweight after controlling for covariates.

Our results suggest a similar psychological distress between underweight and obese. When comparing the charts of underweight and obese participants (Figure 2), we observe a parallel profile, although there is a clear variation in interpersonal sensitivity dimension for both men and women (*p* < 0.001). This dimension focuses on feelings of personal inadequacy and inferiority. Persons with high levels of interpersonal sensitivity show self-deprecation, feelings of uneasiness and marked discomfort during interpersonal interactions. Underweight participants in our study showed statistically significantly higher levels of interpersonal sensitivity than the other BMI categories. A possible explanation to this difference may be in the relationship between BMI and personality traits. Specifically, Kakizaki *et al.* [39] found an inverse association between underweight and extraversion, and a positive association between overweight and extraversion. This result differs from those published by Petry *et al.* [9], since they found that being underweight was inversely related to two specific anxiety disorders (both related to sociability): social phobia and panic disorder with agoraphobia. Nevertheless, there are studies that found higher levels of interpersonal sensitivity (measured with SCL-90-R) in underweight [40] or in obese women [41] than in women with normal weight. Unfortunately, we have not found studies comparing interpersonal sensitivity (measured with SCL-90-R) between underweight and obese people.

Another relevant result of this work was the different level of psychological distress observed among men and women for each BMI categories. Whereas underweight or obese participants showed no gender differences in psychological distress levels, women with normal weight or overweight showed higher levels of psychological distress than men with normal weight or overweight respectively (Figure 1 and Figure 2). Gender differences in psychological status are well known and, in general terms, women show a worse psychological status than men [4,7,14,18,19,27,42]. Studies that have analyzed psychological status by sex in different BMI categories are in agreement with our results on normal weight and overweight participants. Nevertheless, none of those studies found a similar psychological status between men and women, neither for underweight nor obese people [4,7,14,18]. Our finding implicates that having an extreme weight (underweight or obesity) may involves the same psychological danger for both men and women in a Mediterranean population.

Compared with the Spanish normative SCL-90-R scores [27], participants with unhealthy BMI (*i.e.*, underweight, overweight or obese) showed statistically significant higher SCL-90-R adjusted mean scores in all dimensions and GSI (data not shown). Focusing on extreme weight (underweight or obese), the highest discrepancy with norm values was found in phobic anxiety and psychoticism dimensions for both men and women (mean scores above the 99th percentile) [27]. Improving this pathological status of people with extreme weight, will not only benefit to them, but will also mitigate the excess health service use among underweight and obese [43].

Although our study established significant associations between psychological distress and BMI, there were several limitations for this study. First, information about psychological distress was self-reported, and, thus subject to recall bias. Second, although we have adjusted for socio-demographic covariates, we did not have information on other conditions related to BMI which may have affected the associations between BMI and psychological distress. Third, the direction of causality between BMI and psychological distress could not be inferred because of the cross-sectional nature of our study. However, there is enough evidence in order to think that the relationship between BMI and mental health can be bidirectional. [3,44]. Finally, discriminant validity and factorial structure of SCL-90-R has been criticized in several studies [45,46,47,48]. Authors who doubt the validity of SCL-90-R, recommend its use as a measure of general distress instead of interpreting the nine dimensions as independent subscales.

Despite these limitations, strengths of this study are the non-proportional stratified sample in order to include enough participants in all BMI categories, and the use of the psychological scale SCL-90-R, which assess a broad range of psychopathological symptoms.

## 5. Conclusions

We conclude that our findings suggest a symmetric U-shaped relationship between BMI and psychological distress. In our sample, obese and underweight participants showed the same psychological status with the exception of interpersonal sensitivity, so, independently of the direction, the more we move away from the normal weight, the worse the psychological status. Further, contrary to what we expected, we found no gender differences in psychological distress levels for underweight or obese participants. Future studies are needed to confirm this point.

Psychological treatment of Mediterranean people with extreme weight, should consider underweight and obese patients at the same level of psychological distress.
